# Human Adenovirus 36 Antibodies in Horses with Different Metabolic Statuses

**DOI:** 10.3390/ani15172527

**Published:** 2025-08-27

**Authors:** Aleksandra Chwirot, Artur Niedźwiedź, Dominika Stygar, Natalia Siwińska, Marzena Paszkowska, Wojciech Niżański, Skarlet Napierkowska, Paweł Migdał, Agata Kublicka, Maja Marynowska, Anna Matczuk, Devon Fuller, Barbara Bażanów

**Affiliations:** 1 Division of Microbiology, Department of Pathology, Faculty of Veterinary Medicine, Wrocław University of Environmental and Life Sciences, C.K. Norwida 31 Street, 50-375 Wrocław, Poland; aleksandra.chwirot@upwr.edu.pl (A.C.); agata.kublicka@upwr.edu.pl (A.K.); maja.marynowska@upwr.edu.pl (M.M.); anna.matczuk@upwr.edu.pl (A.M.); devon.ksfuller@gmail.com (D.F.); 2 Department of Internal Diseases with Clinic for Horses, Dogs, and Cats, Wrocław University of Environmental and Life Sciences, pl. Grunwaldzki 47, 50-366 Wrocław, Poland; artur.niedzwiedz@upwr.edu.pl (A.N.); natalia.siwinska@upwr.edu.pl (N.S.); 3 Department of Physiology, Faculty of Medical Sciences in Zabrze, Medical University of Silesia, Jordana 19 Street, 41-808 Zabrze, Poland; dstygar@sum.edu.pl; 4 Vetlab, Polish Veterinary Laboratories, 52-017 Wrocław, Poland; marzena.paszkowska@vetlab.pl; 5 Department of Reproduction and Clinic of Farm Animals, Wrocław University of Environmental and Life Sciences, pl. Grunwaldzki 49 Street, 50-366 Wrocław, Poland; wojciech.nizanski@upwr.edu.pl (W.N.); skarlet.napierkowska@upwr.edu.pl (S.N.); 6 Department of Bees Breeding, Institute of Animal Husbandry, Wrocław University of Environmental and Life Sciences, Chełmońskiego 38 Street, 51-630 Wrocław, Poland; pawel.migdal@upwr.edu.pl

**Keywords:** equine virus infection, infectious disease, metabolic status, human adenovirus 36, obesity

## Abstract

This study investigated the presence of antibodies to human adenovirus type 36 (HAdV-D36) in horses with different metabolic statuses, including normal, overweight, and those diagnosed with equine metabolic syndrome (EMS). A total of 151 serum samples were tested, of which 47.6% were positive for anti-HAdV-D36 antibodies. Although the horses were confirmed to be susceptible to HAdV-D36 infection, there was no significant correlation between infection and blood glucose or cholesterol levels. However, the triglyceride levels showed significant differences—they were particularly elevated in the seropositive horses with EMS. These findings suggest that the virus may act by a different mechanism in horses than in other species, and highlight the need for further research to understand its role in horses.

## Simple Summary

The study investigated the presence of anti-human adenovirus 36 (HAdV-D36) antibodies in horses with different metabolic status. Almost half of all the tested subjects were seropositive. No association was found between infection and glucose or cholesterol levels. These results suggest a potentially different mechanism of virus action in horses compared to other species. In the case of triglyceride levels, significantly higher results were observed in the seropositive horses with equine metabolic syndrome (EMS).

## 1. Introduction

Human adenovirus 36 (HAdV-D36) is a non-enveloped virus containing double-stranded DNA (dsDNA) as its genetic material. It belongs to group D of the family *Adenoviridae* and was first isolated in 1978 from a child with diabetes [1]. Since the onset of research on HAdV-D36, the *Adenoviridae* family, traditionally linked to respiratory, gastrointestinal, and ocular infections, has increasingly been associated with obesity, following a growing number of reports implicating this virus in its development [2,3]. The adipogenic potential of adenoviruses has been explored since the 1990s, when the pro-obesity effect of avian adenovirus SMAM-1 in chickens was first observed [4]. Other adenoviruses, such as adenovirus 5 and 37, have also demonstrated the ability to induce obesity in their animal hosts; however, only adenovirus 36 has been shown to infect both humans and animals. The research has confirmed its capacity to infect a variety of species, including mice; rats, hamsters; chickens; dogs; and primates, such as marmosets and rhesus monkeys [5,6,7,8,9].

HAdV-D36 exerts significant influence on gene expression, particularly in the pathways regulating metabolic processes, including the differentiation of pre-adipocytes into adipocytes, enhanced glucose and lipid uptake, and changes in insulin sensitivity [10].

One of the distinguishing features of HAdV-D36 is its unique structure in the protruding region of the hexon protein, which prevents cross-reactivity with antibodies from other adenoviruses [11]. This allows for highly accurate serological studies, facilitating the global screening of different human populations for the presence of HAdV-D36 and its potential link to obesity.

Animal studies have shown that infection with human adenovirus 36 leads to an increase in body weight, mainly by increasing the volume of the fat pads and visceral adipose tissue. Animal models, including rats, adult chickens, and monkeys, have shown that infection with HAdV-D36 leads to an increase in body mass of 15–30% [12,13]. Additionally, HAdV-D36 has been shown to be readily transmitted between infected and uninfected animals via droplet and blood-borne routes [14].

The global rise in the prevalence of antibodies against HAdV-D36 in humans and the results of animal studies underscore the potential significance of this virus in the development of infectious obesity, not only in humans but also in animals [9,10]. Given the virus’s broad host range and ability to infect various species, our research team has explored its seroprevalence in companion animals. Our findings confirmed the ability of HAdV-D36 to infect dogs, a previously undocumented host [9]. We have also included horses in our investigations, with the goal of advancing our understanding of HAdV-D36 and its implications for both human and veterinary medicine.

In our research we include horses with equine metabolic syndrome (EMS), which is our focus, because of the association of human adenovirus 36 with improved insulin sensitivity and glucose metabolism in both animal models and human studies [15]. Equine metabolic syndrome is an endocrine and metabolic disorder characterized by insulin dysregulation, general or regional adiposity, and an increased risk of laminitis. EMS shares some features with human metabolic syndrome, such as increased adiposity and insulin dysregulation [16]. In vitro studies have shown that Ad-36 increases glucose uptake in human skeletal muscle cells from both diabetic and non-diabetic subjects, independent of insulin signaling [17]; this effect is mediated by the Ras-activated PI3K pathway [10,18]. Natural Ad-36 infection has been associated with lower fasting glucose levels in non-diabetics and lower A1Cs in diabetics, suggesting potential therapeutic applications for Ad-36 proteins in the treatment of insulin resistance [18]. Previous adenovirus 36 infection has been associated with greater adiposity, but better glycemic control over time [19].

Human adenovirus 36 (HAdV-D36) is linked to obesity, influencing metabolic pathways and promoting adiposity, while potentially enhancing insulin sensitivity and glucose metabolism. Global serological studies have indicated a significant prevalence of HAdV-D36 in human populations, particularly in children, highlighting its role in “infectious obesity,” and recent research has shown it can also infect animals closely associated with humans, such as dogs and potentially horses [7,9,20]. Comparative studies of horses with different metabolic statuses can provide us with valuable knowledge not only about the host spectrum of human adenovirus 36, but also about the effect of infection on the metabolic status of seropositive animals.

## 2. Materials and Methods

### 2.1. Ethical Statement and Permissions

The samples of serum used in these experiments were taken from horses by veterinarians as a part of annual laboratory blood assessments. In light of this fact, the approval of the Local Ethical Committee was not required. Informed consent concerning use of residues of the clinical specimens for research were obtained from the owners of animals.

### 2.2. Studied Animals and Blood Sampling

The horses included in the study came from various independent and stud farms in the Lower Silesian Voivodeship, Poland. The animals were kept under different conditions, including various feeding systems and types of husbandries, such as a box-paddock system, pasture husbandry, and a mixed system, as well as varying levels of physical activity. The horses within each study group were fed disparate diets. All the animals had ad libitum access to hay and grazing on pasture with variable grazing durations throughout the day. Additionally, some horses received concentrate feed, consisting of commercially prepared mixtures, with the specific type of concentrate tailored to their individual nutritional requirements. Furthermore, the horses tested within each group exhibited varying levels of physical activity, with some classified as sport horses, while others were pleasure or unused horses.

Blood samples were obtained from 151 horses representing diverse breeds, sexes, and metabolic conditions, including normal and disrupted body condition scores, and those diagnosed with equine metabolic syndrome. The samples were drawn from the main jugular vein and collected in tubes containing a coagulant, subsequently centrifuged at 1800 rpm for 10 min to obtain the serum, and prepared for biochemical and serological analyses. Prior to testing, the samples were stored at −80 °C and processed in batches.

### 2.3. Study Group

A total of 151 samples were analyzed and classified into three groups according to the metabolic status of the horses and their body condition score (BCS). The BCS was assessed using the nine-point scale described by Henneke et al., based on a visual inspection and palpation of fat cover at six anatomical sites (tailhead, dorsal midline, withers, neck, ribs, and behind the shoulders) [21].

Horses with a BCS between 4.5 and 5.5 (BCS 4.5–5.5), n = 52.

Horses with a BCS (body condition score, 9-point grade scale) higher than 5.5 (BCS > 5.5), n = 52.

Horses with diagnosed equine metabolic syndrome (EMS), n = 47.

Equine metabolic syndrome was diagnosed on the basis of resting serum insulin concentration 1 h after a meal. Blood samples were collected from the external jugular vein into tubes with a clot activator. After approximately one hour, the samples underwent centrifugation, and the resulting serum was transferred to an Eppendorf tube and cooled. The cooled serum was then analyzed using a commercial radioimmunoassay (RIA) for human insulin (EMD Millipore Corp, Billerica, MA, USA), validated for equine serum. The selection criteria for EMS with ID group required a resting insulin concentration greater than 50 µU/mL.

### 2.4. Virus Neutralization Assay

For preparation of test virus, A549 cells (ATCC-CRM-CCL-185, Manassas, VA, USA) were cultivated in a 175 cm^3^ flask (NEST Scientific Biotechnology, Woodbridge, NJ, USA) with Eagle’s Minimum Essential Medium with Earle’s BSS (Biological Industries, Beit Haemek, Israel) and 10% fetal bovine serum (FBS) (Sartorius, Gottingen, Germany). HAdV-D36 (ATCC VR1610^TM^, Manassas, VA, USA) was added to the monolayer for 2 h at 37 °C with gentle shaking every 15 min. After the cells showed cytopathic effects, they were frozen (−80 °C) and defrosted 3 times followed by a low-speed centrifugation (10 min, 1500× *g*) in order to sediment cell debris. After aliquoting, a test virus suspension was used in the research.

In the first stage, infectivity was assessed as the endpoint titration, transferring 0.1 mL of each dilution into 8 wells of a microtiter plate, beginning with the highest dilution. This was followed by the addition of 0.1 mL of freshly trypsinized A549 cells (10–15 × 10^3^ cells per well). The microtiter plates were incubated at 37 °C in a 5% CO_2_ atmosphere. The plates were observed every day (up to 7 days) and the cytopathic effect was read by using an inverted microscope (ZEISS Axio Observer, Carl Zeiss MicroImaging GmbH, Oberkochen, Germany). Calculation of the infective dose TCID50/mL was calculated with the method of Spearman–Karber with the following formula:

log_10_TCID_50_ = x_0_ − 0.5 + Σr/n

where

x_0_ = log_10_ of lowest dilution with 100% positive reaction;

r = number of positive determinations of lowest dilution step with 100% positive and all higher positive dilution steps;

n = number of determinations for each dilution step.

The presence of antibodies against HAdV-D36 in the tested sera was investigated with a virus neutralization assay on a 96-wells plate. For this purpose, 25 μL of tested serum was mixed with 25 μL of Eagle’s Minimum Essential Medium with Earle’s BSS without any addition and two-fold serial serum dilutions (up to 1:256) were prepared. Then, 25 μL of 100TCID_50_ HAdV-D36 was added. After 1 h incubation, 50 μL of A549 cells were added to each well. For 8 consecutive days the plate was observed using an inverted microscope (ZEISS Axio Observer, Carl Zeiss MicroImaging GmbH, Oberkochen, Germany) to detect the cytopathic effect (CPE).

### 2.5. Biochemical Analysis of Tested Serum

A serum analysis was performed to determine the effect of the virus on the concentrations of glucose, cholesterol, and triglycerides. Samples of frozen serum from the three tested groups were taken sequentially and thawed at 22 °C within 5 min before analysis. Then, 100 microliters of serum was introduced into the chamber of an IDEXX CatalystOne Dx analyzer (Idexx laboratories, Westbrook, ME, USA). The concentration of glucose, cholesterol, and triglycerides in ng/mL was measured sequentially, using Catalyst^TM^ GLU glucose slides, Catalyst^TM^ CHOL cholesterol slides, and Catalyst^TM^ TRIG triglyceride slides (Idexx laboratories, Westbrook, ME, USA). According to the manufacturer of the putters used, the reference ranges are as follows: glucose, 64–150 mg/dL; cholesterol, 50–110 mg/dL; and triglycerides, 11–68 mg/dL.

### 2.6. Statistical Analysis

The statistical analyses were performed using the R program (R-3.4.4 for Windows, CRAN, Vienna, Austria). The normality of data distribution was tested by the Shapiro–Wilk test, while the Kruskal–Wallis test with Holm correction was applied for multiple comparisons to check the differences between groups. For all the tests, a significance level of α = 0.05 was used.

## 3. Results

### 3.1. Virus Neutralization Assay

Among the 151 tested samples of horse serum, 72 (47.7%) samples were positive for the anti-HAdV-D36 antibody and 79 (52.3%) were negative. The detailed data are presented in Table 1. The interpretation proposed by Dhurandhar et al. [22] suggests that every antibody titer higher than or equal to 1:8 is considered a positive test result.

The highest titer of positive anti-HAdV-D36 antibodies was observed in the metabolic syndrome group of horses (*p*-value < 0.05) and the lowest in the horses in the BCS 4.4–5.5 group.

### 3.2. Biochemical Analysis of Tested Serum

Three biochemical parameters—glucose, cholesterol, and triglycerides concentrations—were tested in the obtained horses’ serum, both those seropositive and seronegative for anti-HAdV-D36 antibodies.

#### 3.2.1. Glucose Concentration in Horse Serum

**Figure 1:**
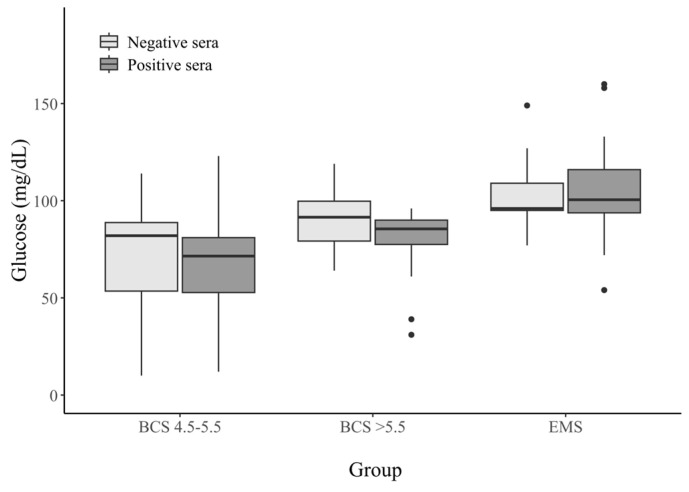
Glucose concentrations (mg/dL) in the sera from horses with different metabolic statuses, stratified by HAdV−D36 serostatus. Dots on the graph—these are outliers.

Significant differences in the glucose levels were observed between the horses with EMS (both seropositive and seronegative) and the BCS 4.5–5.5 and BCS > 5.5 groups; the former had significantly elevated glucose levels (*p*-value ≤ 0.05). A similar difference was observed between the group of obese horses and those with a BCS higher than 5.5. The obese horses presented with elevated levels of glucose concentration. Furthermore, between the horses with a BCS of 4.5–5.5 and those with a BCS > 5.5, those that were seropositive had slightly lower glucose levels than the seronegative individuals. The detailed results are presented in Table 2 and in Figure 1.

#### 3.2.2. Cholesterol Concentration in Horse Serum

**Figure 2:**
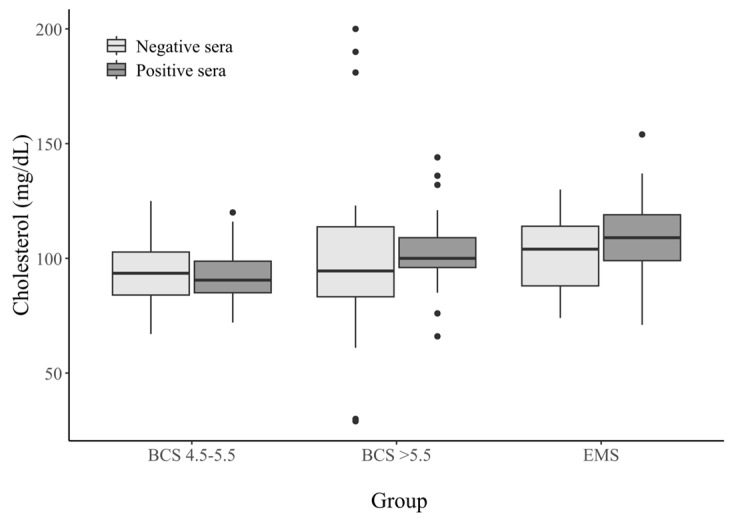
Cholesterol concentrations (mg/dL) in the sera from horses with different metabolic statuses, stratified by HAdV−D36 serostatus. Dots on the graph—these are outliers.

There was a statistically significant difference in the cholesterol levels between the BCS 4.5–5.5 group (both positive and negative) and the other groups (*p*-value < 0.05). These horses had slightly lower cholesterol levels. For this parameter we did not observe differences in the concentration between the seropositive and seronegative horses in any of the study groups. The data are shown in Table 3 and Figure 2.

#### 3.2.3. Triglycerides Concentration in Horse Serum

**Figure 3:**
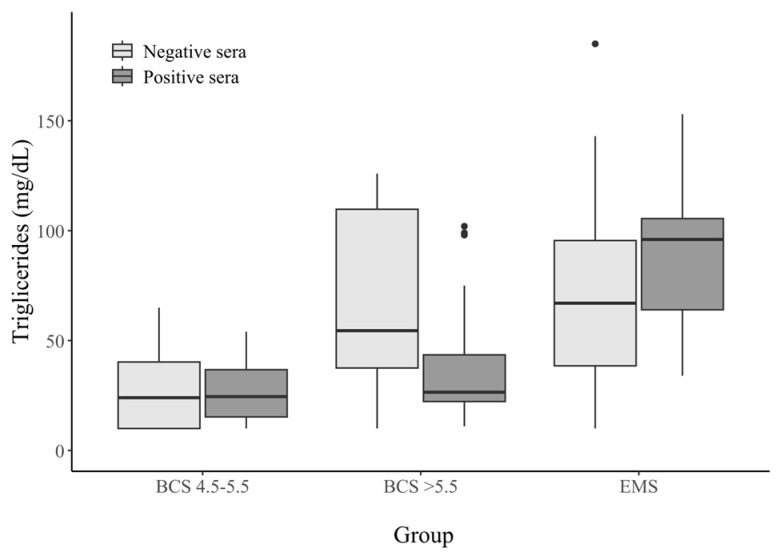
Triglycerides concentrations (mg/dL) in the sera from horses with different metabolic statuses, stratified by HAdV−D36 serostatus. Dots on the graph—these are outliers.

We did not observe a difference in the concentration of this parameter in the BCS 4.5–5.5 horse group (*p*-value < 0.05). In the group of horses with a BCS > 5.5, significantly lower triglyceride concentrations were observed among the seropositive horses than the seronegative horses. The serum triglyceride concentrations in the seropositive EMS horses were significantly higher than those observed in the samples from the other groups. The results are shown in Table 4 and in Figure 3.

## 4. Discussion

In our study, we tested the sera of 151 horses for the presence of anti-HAdV-D36 antibodies. A total of 47.7% of the sera tested was seropositive. Our study is, therefore, to the best of our knowledge, the first report indicating that horses can be infected with human adenovirus 36.

Various studies have investigated the adipogenic effects of HAdV-D36 in humans; monkeys; chickens; dogs; and model animals, such as mice and rats. They have provided valuable insights into the virus’s mechanism of action and the associated differences in cholesterol, triglycerides, and glucose levels in infected individuals [9,12,20,23,24]. Na et al. showed that HAdV-D36 infection in mice causes obesity by inducing inflammatory responses and raising MCP-1 (macrophage chemotactic factor) levels [25]. Researchers have confirmed that HAdV-D36 infection promotes adipogenesis, induces pro-inflammatory effects within adipose tissue, increases cellular glucose uptake, and impairs endocrine metabolism, leading to the accumulation of glucose in muscle cells and cholesterol and triglycerides in adipocytes [17,23]. Additionally, HAdV-D36 infection can increase appetite in infected individuals by reducing the levels of norepinephrine and leptin, both of which are responsible for regulating satiety and hunger [10]. While the results of these studies were relatively consistent in humans and the aforementioned animal species, thus confirming the previously proposed mechanism of action, the discovery of a broader host range for HAdV-D36 has complicated the interpretation of these mechanisms. In our earlier studies on dogs, in which we confirmed their susceptibility to infection and successfully isolated the virus from canine adipose tissue, the blood parameters measured did not align with the patterns previously observed in humans, monkeys, chickens, and rodents. In contrast to mice and chickens, where a significant decrease in cholesterol levels was observed following infection, no such correlation between cholesterol reduction and the presence of anti-adenovirus 36 antibodies was detected in dogs [4,9]. Similar results were obtained in this study, where we examined horses with different weights and metabolic statuses. Our results indicate that horses can be infected with human adenovirus 36; however, unlike in other animal species, infection was not associated with serum glucose or cholesterol levels. A positive correlation was observed only between the antibody titers and triglyceride levels. This dependence may be due to the fact that horses are herbivorous. In our research, a statistically significant difference in cholesterol levels was observed between the healthy, normal-weight horses (both seropositive and seronegative) and the BCS > 5.5 and EMS groups. This is the first research to be conducted on the seroprevalence of human adenovirus 36 in horses.

Within the BCS > 5.5 horse group, the presence of anti-HAdV-D36 antibodies was also not a significant factor influencing the cholesterol serum concentration. Furthermore, the seronegative obese horses had lower cholesterol concentrations than the seropositive horses diagnosed with equine metabolic syndrome (EMS), which tended to exhibit higher cholesterol levels, which aligns with the metabolic disturbances observed in other species suffering from metabolic syndrome [16]. These results suggest that the observed differences in cholesterol levels among the tested horses are likely attributable to species-specific physiology rather than to HAdV-D36 infection. This could be linked to insulin dysregulation, altered lipid metabolism, and systemic inflammation, which are common features of EMS. In contrast, the healthy horses maintained lower cholesterol concentrations, likely due to more efficient lipid homeostasis and metabolic regulation [26,27]. Similar correlation coefficients of this parameter with anti-AdV-D36 antibody levels were observed in dogs. Seropositive dogs did not show reduced serum cholesterol levels [9]. In contrast to mice, where HAdV-D36 infection reduced cholesterol [22], we observed no association between seropositivity and cholesterol in horses.

The situation is markedly different with regard to triglycerides. In this case, according to Dhurandhar’s studies on experimental animals (rhesus, marmosets, mice, and chickens), an increase in anti-HAdV-D36 antibodies is accompanied by a decrease in triglyceride levels [22,28]. Similar observations have also been reported in dogs [9]. In horses, however, the relationship between triglyceride levels and HAdV-D36 serostatus appears to depend on the physiological condition of the animals. Among obese horses, seropositive individuals exhibited triglyceride concentrations approximately half those of seronegative individuals, consistent with the findings in other species [22,28]. However, this difference was not confirmed in the BCS 4.5–5.5 horse group. The highest statistically significant TG levels across all the horses were recorded among seropositive individuals diagnosed with metabolic syndrome. Nevertheless, as with cholesterol, these findings may reflect the specific characteristics of the disease rather than being directly attributable to viral infection. In horses with equine metabolic syndrome (EMS), elevated triglyceride concentrations are a well-documented hallmark of the underlying insulin dysregulation and altered lipid metabolism, which are core features of the syndrome. These animals frequently exhibit abnormal fat deposition and reduced insulin sensitivity, both of which contribute to impaired lipid clearance from the bloodstream. Therefore, the increased TG levels observed in seropositive horses may not be a consequence of viral exposure per se, but rather an expression of the metabolic disturbances inherent to EMS. As far as humans are concerned, Barrera-Alcocer et al. and Ponterio et al. did not demonstrate a similar correlation between HAdV-D36 seropositivity and triglyceride levels. They both tested levels of different parameters (including triglycerides) in serum of patients with the presence of HAdV-D36 confirmed with a PCR [29,30]. This discrepancy suggests that adenovirus 36 may act through a different mechanism in humans compared to animals. Another parameter that has been investigated is blood glucose. From the data available in the literature, it is known that HAdV-D36 infection significantly reduces blood glucose levels in humans, rats, and rhesus monkeys [27,28]. Shirani et al. found that the HAdV-D36 virus did not induce significant changes in inflammatory markers, such as TNF-α (tumor necrosis factor-alpha), IL-6 (interleukin-6), and MCP-1. They did, however, observe significant improvements in the fasting glucose and insulin parameters, increased insulin sensitivity, and reduced total cholesterol and triglyceride levels in infected animals compared to the controls, thus confirming the findings of other researchers [31]. Pasarica et al. confirmed that rats infected with HAdV-D36 have increased insulin sensitivity and glucose uptake [12]. Kapila et al. and Dhurandrah et al. in their research showed that an increase in weight is accompanied by a decrease in cholesterol levels [14,32]. Improved glycemic control, caused by the virus, has been attributed to increased mitochondrial activity in the liver [33] and, as shown in humans, increased glucose uptake by skeletal muscle cells, independent of insulin signaling [17].

Interestingly, in the seropositive horses, we did not observe a reduction in glucose levels associated with the presence of antibodies, which may indicate that the mechanism of action of HAdV-D36 described above does not apply to these animals or that it acts in a different, as yet unidentified way. Although seropositive individuals exhibited lower glucose levels than their seronegative counterparts, these differences were not statistically significant. The glucose levels varied significantly between the different groups of horses. The lowest glucose levels were in the BCS 4.5–5.5 group, slightly higher levels in the BCS > 5.5 group, and the highest in the EMS group. The highest glucose concentrations were observed in the seropositive horses with metabolic syndrome, a finding most likely attributable to the specific pathophysiology of the disease itself. Unlike healthy horses, which typically maintain stable glucose homeostasis through effective insulin regulation, EMS-affected individuals experience insulin dysregulation, leading to persistent hyperglycemia and an increased risk of laminitis and other metabolic complications [34].

The presented study has several limitations. Frozen serum samples were used, but due to the retrospective nature of the study, it was not possible to use freshly collected sera. However, we believe that freezing at −80 °C did not significantly affect the results of our study. Shortly after collecting the sera, the samples were divided from the blood and frozen at a temperature of −80 °C to prevent any physiological processes that could have distorted the results [35]. Another limitation of this study was the inability to evaluate the influence of diet and exercise levels on the test outcomes within each group. Given the considerable variability in the dietary regimens among the studied horses, such an assessment was not feasible. Additionally, it appears unlikely that the viral infection was diet-dependent.

In summary, the fluctuations in the blood parameters associated with HAdV-D36 infection in humans and other animals do not correspond exactly to the observations made in horses. It is unclear at this stage whether this discrepancy is due to distinct viral mechanisms related to the unique physiology of horses, or to the specificity of the infecting strain. Further studies involving a larger number of individuals, conducted at specific time intervals, are needed to clarify these discrepancies. Studies conducted under more controlled conditions could provide a clearer picture of the virus’s biology and its impact on equine organisms.

## 5. Conclusions

Our study confirms that horses are susceptible to HAdV-D36 infection (47.7% seropositivity) and there is a unique association between seropositivity and elevated triglycerides in EMS horses, distinct from the patterns seen in humans and other animals. Further research into equine-specific viral mechanisms and transmission is warranted.

## Figures and Tables

**Table 1:** Virus neutralization test results for HAdV-D36 antibodies in serum of tested horses.

SN Antibody Titer	BCS 4.5–5.5 (n = 52)	BCS > 5.5 (n = 52)	EMS (n = 47)	
0	19	22	16	Seronegative (n = 79, 52.3%)
1:2	5	3	0	
1:4	6	2	6	
1:8	8	4	6	Seropositive (n = 72, 47.7%)
1:16	8	3	7	
1:32	1	9	4	
1:64	4	6	5	
1:128	0	2	1	
1:256	1	1	0	

**Table 2:** Glucose concentrations (mg/dL) in the sera from horses with different metabolic statuses, stratified by HAdV-D36 serostatus.

Glucose (mg/dL)	BCS 4.5–5.5		BCS > 5.5		EMS	
	Seropositive Sera (n = 22)	Seronegative Sera (n = 30)	Seropositive Sera (n = 25)	Seronegative Sera (n = 27)	Seropositive Sera (n = 25)	Seronegative Sera (n = 21)
0–50	5 (22.7%)	7 (23.3%)	2 (8%)	0 (0%)	0 (0%)	0 (0%)
51–64	3 (13.6%)	2 (6.6%)	1 (4%)	2 (7.4%)	1 (4%)	0 (0%)
65–84	9 (40.9%)	11 (36.6%)	7 (28%)	9 (33.3%)	2 (8%)	2 (9.5%)
85–100	3 (13.6%)	7 (23.3%)	15 (60%)	11 (40.7%)	9 (36%)	9 (42.8%)
101–125	2 (9%)	3 (10%)	0 (0%)	5 (18.5%)	9 (36%)	8 (38%)
126–150	0 (0%)	0 (0%)	0 (0%)	0 (0%)	1 (4%)	2 (9.5%)
>151	0 (0%)	0 (0%)	0 (0%)	0 (0%)	3 (12%)	0 (0%)

**Table 3:** Cholesterol concentrations (mg/dL) in the sera from horses with different metabolic statuses, stratified by HAdV-D36 serostatus.

Cholesterol (mg/dL)	BCS 4.5–5.5		BCS > 5.5		EMS	
	Seropositive Sera (n = 22)	Seronegative Sera (n = 30)	Seropositive Sera (n = 25)	Seronegative Sera (n = 27)	Seropositive Sera (n = 25)	Seronegative Sera (n = 21)
0–50	0 (0%)	0 (0%)	0 (0%)	2 (7.4%)	0 (0%)	3 (14.2%)
51–80	5 (22.7%)	7 (23.3%)	1 (4%)	5 (18.5%)	1 (4%)	2 (9.5%)
81–90	6 (27.2%)	6 (20%)	2 (8%)	6 (22.2%)	2 (8%)	5 (23.8%)
91–110	9 (40.9%)	14 (46.6%)	16 (64%)	6 (22.2%)	16 (64%)	5 (23.8%)
>111	0 (0%)	3 (10%)	6 (24%)	8 (29.6%)	6 (24%)	12 (44.4%)

**Table 4:** Triglycerides concentrations (mg/dL) in the sera from horses with different metabolic statuses, stratified by HAdV-D36 serostatus.

Triglycerides (mg/dL)	BCS 4.5–5.5		BCS > 5.5		EMS	
	Seropositive Sera (n = 22)	Seronegative Sera (n = 30)	Seropositive Sera (n = 25)	Seronegative Sera (n = 27)	Seropositive Sera (n = 25)	Seronegative Sera (n = 21)
0–11	4 (18.1%)	9 (30%)	1 (4%)	3 (11.1%)	0 (0%)	1 (4.7%)
12–30	8 (36.3%)	9 (30%)	15 (60%)	2 (7.4%)	0 (0%)	3 (14.2%)
31–50	7 (31.8%)	10 (33.3%)	5 (20%)	5 (18.5%)	4 (16%)	3 (14.2%)
50–68	1 (4.5%)	2 (6.6%)	1 (4%)	5 (18.5%)	4 (16%)	4 (19%)
>69	2 (9%)	0 (0%)	3 (12%)	12 (44.4%)	17 (68%)	8 (38%)

## Data Availability

The datasets used and/or analyzed during the current study are available from the corresponding author on reasonable request.
